# Survival, Incidence, and Mortality Trends in Female Cancers in the Nordic Countries

**DOI:** 10.1155/2023/6909414

**Published:** 2023-07-07

**Authors:** Filip Tichanek, Asta Försti, Otto Hemminki, Akseli Hemminki, Kari Hemminki

**Affiliations:** ^1^Biomedical Center, Faculty of Medicine, Charles University Pilsen, Pilsen 30605, Czech Republic; ^2^Hopp Children's Cancer Center (KiTZ), Heidelberg, Germany; ^3^Division of Pediatric Neurooncology, German Cancer Research Center (DKFZ), German Cancer Consortium (DKTK), Heidelberg, Germany; ^4^Cancer Gene Therapy Group, Translational Immunology Research Program, University of Helsinki, Helsinki, Finland; ^5^Department of Urology, Helsinki University Hospital, Helsinki, Finland; ^6^Comprehensive Cancer Center, Helsinki University Hospital, Helsinki, Finland; ^7^Division of Cancer Epidemiology, German Cancer Research Center (DKFZ), Im Neuenheimer Feld 580, D-69120, Heidelberg, Germany

## Abstract

**Background:**

Female cancers cover common breast cancers, relatively common endometrial, ovarian, and cervical cancers and rare vulvar cancer. Survival in these cancers is known to be relatively good compared to all cancers but long-term studies for these cancers are rare, and to fill the gap, here, we generate survival data through 50 years.

**Materials and Methods:**

We applied generalized additive models to data from the NORDCAN database and analyzed 1- and 5-year relative survival for these cancers in Denmark (DK), Finland (FI), Norway (NO), and Sweden (SE) over half a century (1971–2020). Conditional 5/1-year survival for patients who survived the 1st year after diagnosis and annual survival changes was also estimated.

**Results:**

In 2016–20, 5-year survival was best for breast cancer reaching 92.3% (in SE), followed by endometrial cancer at 86.1% (SE) and cervical cancer at 75.6% (NO). Improvement in 5-year survival over the 50 years was the largest for ovarian cancer (20% units), finally reaching 52.9% (SE). For vulvar cancer, the final survival was between 70 and 73%. The best 5-year survival rate in 2016–20 was recorded for SE in breast, endometrial, and ovarian cancers; NO showed the highest rate for cervical and DK for vulvar cancers. DK had the lowest survival for breast and ovarian cancers, and FI, for the other cancers.

**Conclusions:**

The overall survival development appeared to consist of continuous improvements, most likely because of novel treatment and imaging techniques as well as overall organization of patient care. The large survival improvement for ovarian cancer was probably achieved by a surgical focus on tumors spread in the peritoneal cavity. For cervical and vulvar cancers, the high early mortality requires attention and could be helped by raising increasing public awareness of early symptoms in these cancers and developing pathways for fast initiation of treatment.

## 1. Introduction

In developed countries, the prevalence of female cancers ranges from the most common breast cancer to endometrial cancer, to ovarian and cervical cancers, and to the rare vulvar cancer. Modifiable risk factors for these cancers differ extensively; population attributable fractions (PAFs) are estimated at 70% or higher for human papilloma virus (HPV) infections for cervical and lower for vulvar cancer [1, 2]. Obesity is an important risk factor for endometrial cancer and a minor risk factor for breast and ovarian cancers; postmenopausal hormone therapy is another risk factor for endometrial and also for breast cancer [1, 2]. For breast cancer, smoking, alcohol drinking, and the absence of breastfeeding increase the risk [1, 2]. For breast, endometrial and ovarian cancers reproductive factors, low parity, and late first childbirth are risk factors, but these are not usually included as modifiable risk factors [3–7]. Family history is a risk factor for all female cancers [8]. The total number of ovulation cycles is associated with the risk of ovarian and endometrial cancers [9]. Oral contraception is protective of these cancers but may increase the risk of breast cancer [10–12]. It has been estimated that a part of the past increase in the incidence of breast cancer can be explained by changing reproductive factors, such as low parity, and by increasing obesity [13].

Population screening has been organized in the Nordic countries for cervical and breast cancers. National cervical cancer screening programs were implemented in Finland (FI) in 1971, Sweden (SE) in 1973, Norway (NO) in 1995, and Denmark (DK) in 1996, and attendance rates have been generally high [14, 15]. In NO and DK, regional screening was started even before the national one [14, 16]. As a result of the screening, the incidence trends for cervical cancer decreased markedly [17, 18]. Implementation of national breast cancer screening started in FI and SE in 1986. DK initiated the implementation in 1991 and in NO in 1996 [19–22]. Survival in female cancers has generally improved in the Nordic countries and elsewhere but the exact reasons have remained unclear as many factors influence survival, ranging from demographics (age, sex, social background) and cancer-related (stage, grade) factors to diagnostics, treatment, and overall patient care [23–28].

We assess here relative survival in female cancers in the above four Nordic countries through 50 years up to 2020 based on the NORDCAN database. We apply three relative survival metrics, 1-, 5- and conditional 5/1-year survival to allow the focus on the country-specific changes in survival. The countries have organized health care largely in a similar way and with a principle of access with minimal direct out-of-pocket costs to patients. The Nordic cancer registries are the oldest national cancer registries in the world and they cover practically all cancers without loss to follow-up [29].

## 2. Methods

The data were obtained from NORDCAN database 2.0, assessed in the fall of 2022 [29, 30]. The database was accessed at the International Agency for Cancer (IARC) website (https://nordcan.iarc.fr/en) [31], and the available tools were used to extract data for a cohort study on incidence, mortality and 1- and 5-year survival. International Classification of Diseases (ICD) version 10 codes were used in NORDCAN to describe the tumor locations. The code for breast cancer was C50, endometrial cancer C54, ovarian cancer C56, C57.0–C57.4 (including also tubes), cervical cancer C53, and vulvar cancer C51.

The follow-up was terminated at death, emigration or loss of follow-up or by the end of 2020. Incidence and mortality data were age-standardized for the world standard population. For incidence and mortality data, the starting date was 1961 (the earliest available for all countries). Survival data for relative survival were available from 1971 onwards and the analysis was based on the cohort survival method for the first nine 5-year periods, and a hybrid analysis combining period and cohort survival in the last period 2016–2020, as detailed [32, 33]. Age-standardized relative survival was estimated using the Pohar Perme estimator [34]. Age standardization was performed by weighting individual observations using external weights as defined on the IARC website. Age groups 0 to 89 were considered. The DK, FI, NO and SE life tables were used to calculate the expected survival. All survival data were recorded in 5-year periods with 95% confidence intervals (95% CIs) which allow the assessment of significant periodic improvements in survival and comparisons between the sexes and countries.

Statistical modelling and data visualizations were performed using *R* statistical software (https://www.r-project.org) in the *R* studio environment (https://posit.co/) (code available at https://github.com/filip-tichanek/nord_female) [35]. Time trends of 1- and 5-year relative survival (in %; obtained from NORDCAN for each of the 5-year periods) were modelled using the Gaussian generalized additive models (GAM) as detailed [35]. The GAM model included the effect of *country* and *country*-specific nonlinear effect of *time* and were run in the Bayesian framework using the “brms” *R* package [36, 37], which employs “Stan” software for probabilistic sampling [38]. For the 5/1-year conditional survival ratio estimation, we divided the posterior draws from the 5-year survival model by the posterior draws from the 1-year model to get the posterior distribution of the conditional survival and its estimated annual changes over time [35]. This conditional survival gives survival probability for patients who had survived the first year after diagnosis to survive to year 5.

For all survival measures (relative 1-, 5-year and 5/1-year conditional), we evaluated when the survival was changing over time with at least 95% plausibility (95% credible interval [Ci] of the 1st derivation of given survival measure did not cross zero for at least 5 years) [35]. We also aimed to identify “breakpoints,” i.e. times when the annual change of survival changed with at least 95% plausibility. This was assessed by calculation of the 2nd derivation of the given survival measure and its 95% Ci; the “breakpoint” was defined as a peak value within at least a 3-year interval where 95% Ci for the 2^nd^ derivation did not cross zero.

Comparisons with the US Surveillance, Epidemiology and End Results (SEER) data for years 2012–18 on Non-Hispanic white women were done through (https://seer.cancer.gov/statistics-network/explorer/application.html?site=1&data_type=1&graph_type=2&compareBy=sex&chk_sex_3=3&chk_sex_2=2&rate_type=2&race=1&age_range=1&hdn_stage=101&advopt_precision=1&advopt_show_ci=on&hdn_view=0&advopt_display=2#graphArea).

## 3. Results

### 3.1. Incidence and Mortality in the Nordic Countries

**Figure 1:**
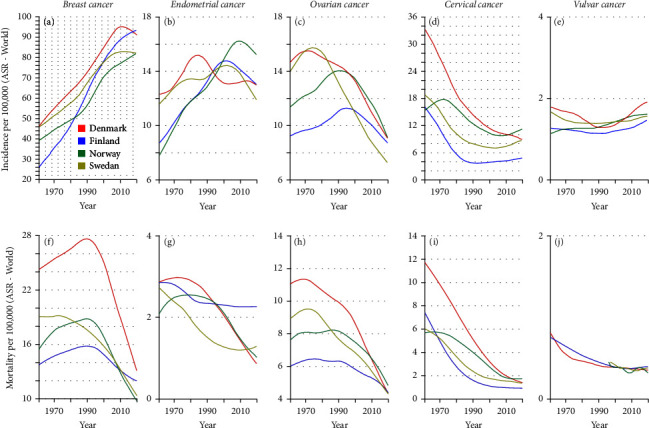
Incidence (a–e) and mortality (f–j) of breast (a, f), endometrial (b, g), ovarian cancer (c, h), cervical cancer (d, i), and vulvar cancer (e, j). Lines were smoothed via cubic smoothing spline.

Age-standardized (world) incidence and mortality trends for the five female cancers from 1961 to 2020 are shown in Figure 1. In the top row, the large incidence differences are apparent between the cancers, with breast cancer peaking at around 90/100,000, and vulvar cancer below 2/100,000. DK has the highest incidence and mortality rates for many of these cancers and FI has the lowest rates.

### 3.2. Relative Survival

**Figure 2:**
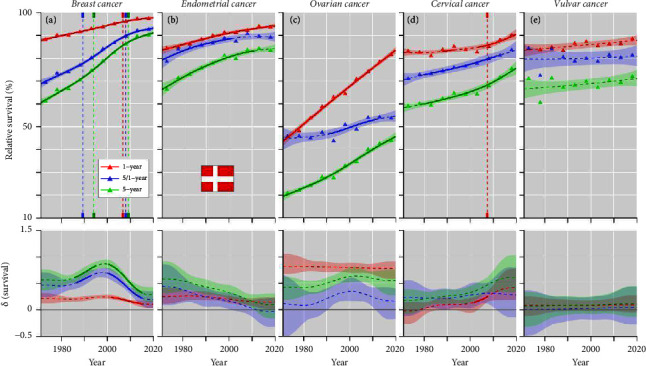
Relative 1-, 5/1- and 5-year survival of female cancers in Denmark, (a) breast, (b) endometrial, (c) ovarian, (d) cervical, and (e) vulvar cancers. The vertical lines mark a detectable change in the survival trends (“breakingpoints”) and the curves in the lower panels show estimated annual changes in survival. The curves are solid if there is >95% plausibility that the curve grows or declines. Shadow areas indicate 95% credible interval derived from GAM. All curves are color coded (see the insert).

Relative survival in the female cancers in DK is shown in Figure 2. For breast cancer, a steady increase was observed, which for 5-year survival started at 60% in 1971–75 and ended at 90% in 2016–20. Annual improvements in survival were highest, close to 1% units at around 2000. For endometrial cancer, both 1- and 5-year survival improved but less than those for breast cancer. For ovarian cancer, with low starting levels, survival graphs increased almost linearly, and 1-year survival increased from 45 to over 80%. For cervical cancer, 5-year survival started at 60%, and, bending upwards, ended at 75%. Survival in vulvar cancer did not change noticeably, with 5-year survival ending at close to 70%.

**Figure 3:**
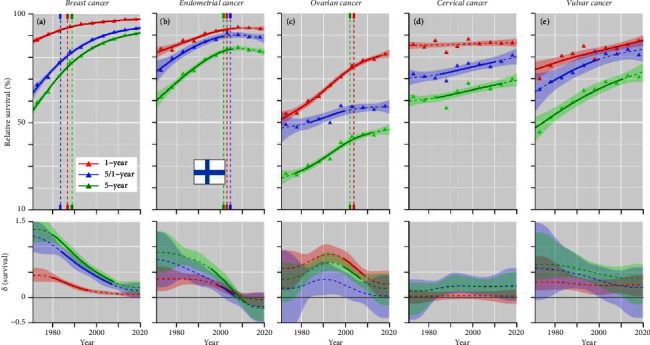
Relative 1-, 5/1- and 5-year survival of female cancers in Finland, (a) breast, (b) endometrial, (c) ovarian, (d) cervical, and (e) vulvar cancers. The vertical lines mark a detectable change in the survival trends (“breakingpoints”) and the curves in the lower panels show estimated annual changes in survival. The curves are solid if there is >95% plausibility that the curve grows or declines. Shadow areas indicate 95% credible interval derived from GAM. All curves are color coded (see the insert).

In FI, the survival patterns were not very different from those in DK, except for vulvar cancer with a low starting level and steady increase till 2010 (Figure 3). For endometrial cancer the positive development culminated in 2000, and for ovarian and cervical cancers all survival curves were below the DK ones.

**Figure 4:**
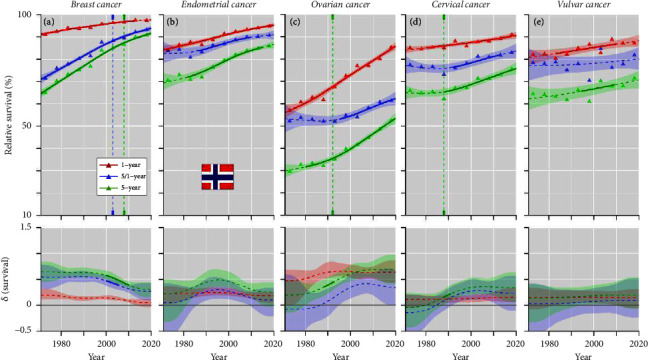
Relative 1-, 5/1- and 5-year survival of female cancers in Norway, (a) breast, (b) endometrial, (c) ovarian, (d) cervical, and (e) vulvar cancers. The vertical lines mark a detectable change in the survival trends (“breakingpoints”) and the curves in the lower panels show estimated annual changes in survival. The curves are solid if there is >95% plausibility that the curve grows or declines. Shadow areas indicate 95% credible interval derived from GAM. All curves are color coded (see the insert).

In NO, survival patterns for the female cancers resembled those for DK and FI, but tended to be better in the starting and finishing levels (Figure 4). Conditional 5/1-year survival curves for ovarian and cervical cancers started to increase first in 2000 which was due to the steep increase in 1-year survival.

**Figure 5:**
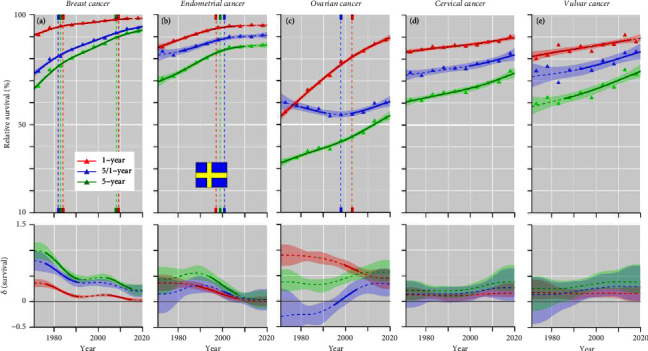
Relative 1-, 5/1- and 5-year survival of female cancers in Sweden, (a) breast, (b) endometrial, (c) ovarian, (d) cervical, and (e) vulvar cancers. The vertical lines mark a detectable change in the survival trends (“breakingpoints”) and the curves in the lower panels show estimated annual changes in survival. The curves are solid if there is >95% plausibility that the curve grows or declines. Shadow areas indicate 95% credible interval derived from GAM. All curves are color coded (see the insert).

In SE, the survival curves were often at the best level of the Nordic countries (Figure 5). Among these achievements, 5-year survival in breast cancer ended at 92.5%, in endometrial cancer at 86.1% and ovarian cancer at 52.9%. For ovarian cancer, 5/1-year survival curve declined up to 2000. Vulvar cancer survival modestly increased from 1985.

Supplementary Table 1 displays a comparison of the survival experience between the countries. The differences were large but overall they declined with time but some significant differences remained. The last 5-year survival rate was highest for SE in breast, endometrial and ovarian cancers; NO showed the highest rate for cervical and DK for vulvar cancer. DK had the lowest survival for breast and ovarian cancers, and FI for the other cancers. The differences between the countries of best and worst survival were significant, except that in cervical cancer no country deviated significantly.

From Figures 2–5 and Supplementary Table 1, one can estimate the magnitude of survival improvements during the 50-year period. Improvement in 1-year survival was about 10% units for all cancers, except for cervical cancer (5% unit) and ovarian cancer (30% units). For 5-year survival breast and ovarian cancer presented an improvement of 20% units or more, endometrial cancer slightly less and the other cancers between 5 and 15% (vulvar cancer improved by 30% units in NO).

## 4. Discussion

At the beginning of the follow-up (1971–75), 5-year survival for female cancers was best for endometrial cancer for which NO and SE had already reached a survival level of more than 70%, some 5% units better than survival in breast cancer. However, it took no more than 15 years for NO and SE breast cancer to catch up the survival rates in endometrial cancer (catching up was slower in DK and FI). In the end, breast cancer survival was the highest of the present cancers, with the best rate of 92.5% for SE. For endometrial cancer, an 80% survival was reached before 2010. Survival in ovarian cancer started at a low level (20% in DK); SE reached 50% by 2011–15 but DK and FI were slower and reached 45% towards the end of the follow-up. Survival in cervical cancer developed well in NO, and the 5-year rate reached 70% already in 2001–05, while for FI that target remained yet to be reached. For vulvar cancer, the final survival was very similar across countries (slightly over 70%). We discuss the five cancers individually below.

The three survival metrics showed that in breast cancer 1-year survival reached 95% level during the 1990s. Also, conditional 5/1-year survival improved steadily indicating that survival also improved in those who had survived the first year of their cancer. Survival improvements in breast cancer have often been assigned to treatment, improving imaging methods and possibly also screening mammography [39, 40]. Surgery has historically been the main therapeutic modality, supported by radiotherapy [41]. Adjuvant therapies were started already in the 1970s with a positive impact on survival; their indications have been widened and novel agents have been taken to use [41]. Chemotherapy and hormone therapy is used in metastatic and recurrent breast cancer [42]. The gradual, even improvement in survival implies the contribution of many factors from the beginning of the follow-up in the 1970s.

According to the global cancer survival study, all Nordic countries reached for breast cancer the top 5-year relative survival rank of 85 to 90%, shared by 25 countries (of a total of 66 countries) for the years 2010–14 [24]. The 9 nonEuropean countries included Costa Rica, Martinique, Canada, US, Israel, Japan, Korea, Australia and New Zealand. Breast cancer survival improved in the Nordic countries since the above international study and 5-year survival reached 90% in 2016–20. The present US SEER data on breast cancer for Non-Hispanic white women for years 2012–18 of 92.0% are on par with the SE 92.3% and slightly better than the other Nordic survival data. For breast cancer, 5-year survival span is quite short as recurrences may occur as late as 30 years after the initial diagnosis. Even patients with metastatic breast cancer currently live beyond 5 years; there are a lot of treatments for metastatic disease but they are not curative.

For endometrial cancer, there were subtle differences in survival metrics after 2000. For NO 1- and 5-year survival kept on increasing while for the other countries these levelled off (Supplementary Table 1). Thus 5-year survival remained 5 or more % units below that of breast cancer. Surgical treatment for endometrial cancer consists of hysterectomy with bilateral salpingo-oophorectomy, sentinel node biopsies, and lymphadenectomy [43]. A majority of patients have a low risk of recurrence and are managed by surgery alone; radiotherapy is additionally recommended for patients with higher risk, and for metastatic patients kinase inhibitors and immunotherapy can be offered [43]. In the US SEER database, for cancer of the “corpus and uterus, NOS” 5-year survival for Non-Hispanic white women for years 2012–18 was 84.1%, not much below the best Nordic survival at 86.1% in SE. A Korean study up to year 2017 reported 5-year relative survival of 89% and it was over 90% for other main histologies but the serous type [44].

Ovarian cancer showed by far the highest survival improvements among the female cancers during the 50-year period. The impressive improvement in 1-year survival by some 30% units scored in the top rank of all solid tumors in the Nordic countries [27]. The rate of increase in 1-year survival was higher than the improvement in 5-year survival, the consequence of which was that conditional 5/1-year survival barely improved (most clearly for SE). This is an example of a survival scenario where improvements in 5-year survival are not able to balance improvements in 1-year survival, thus the declining or relatively constant 5/1-year survival. We have reported similar survival trends for liver and pancreatic cancers for which 1-year survival remarkably increased [45]. Surgery is also the main treatment for ovarian cancer with an aim at removing all suspicious lymph nodes in early stage disease and in radical cytoreductive surgery for removal as much tumor as possible [46]. Notably, ovarian cancer is one of the few tumor types where surgery is routinely preformed despite metastases. Adjuvant chemotherapy may be administered (often paclitaxel and carboplatin with or without bevacizumab) and more recently with PARP inhibitors [46]. Judging from the almost linearly increasing 1-year survival over the 50-years, improvements in therapy and imaging techniques are most like the major contributors to this achievement. In the above global survival study, all Nordic countries were in the top survival group for ovarian cancer (5-year at 45–50%, only Costa Rica had a survival of over 50%) [24]. In the US SEER database ovarian cancer 5-year survival for Non-Hispanic white women for years 2012–18 was 48.5%, below NO (52.4%) and SE (52.9%) but over DK and FI.

The development in cervical cancer resembled that for endometrial cancer but at a somewhat lower level, and the overall improvement in 1- and 5-year survival was a mere 10% units. In the Nordic countries attendance rates in the national cervical cancer screening programs are generally high but one can speculate that a negative screening result may provide false assurance and neglectable of early symptoms. Vulvar cancer showed country-specific trends, particularly a very low early survival in NO. Some 10% of cervical and up to 20% of vulvar cancer patients died within the year of diagnosis even in the last period suggesting that in these cases diagnoses were delayed. We have reported earlier that survival in cervical and vulvar cancer is age-group related and old patients are surviving worst and this has been seen also in an international study [24, 28]. Cervical and vulvar cancers are historically treated with surgery. Multimodal chemoradiotherapy or combining surgery and adjuvant chemoradiation were later therapeutic additions. Chemotherapy is used in advanced tumors either as neoadjuvant or palliative setting [28, 47]. The ongoing vaccination against HPV will eventually offer protection against cervical and vaginal cancers but its full impact on case reduction will require decades [48, 49]. Immunotherapy has shown promising results in metastatic cervical cancer and may offer a bridge towards protection by vaccination [50]. According to the above global cancer survival study, data for cervical cancer were available on 64 countries and 5-year survival reached 70% in 7 countries, including DK and NO, but also Japan, Korea, Taiwan, Switzerland and Cuba [24]. DK and NO were also leading countries in the present study with the final 5-year survival rates of 75.4 and 75.5%. In the above US SEER study, cervical cancer survival was 67.3%, well below the Nordic figures. The SEER data showed survival in vulvar cancer to be 69.6%, what is somewhat below the Nordic countries (70.6 to 72.9%). In a separate study from the SEER database from 2001 to 2011, 5-year survival for cervical cancer was 64.2% and for vulvar cancer of squamous cell histology it was 66% [51]. A Korean study on vulvar cancer covering years 1999 to 2018 showed a constant survival of 74% throughout the period [52].

The limitations in the NORDCAN database are lacking pathological, clinical, and diagnostic data which exclude possibilities to adjust for grade, for example. However, the unique advantages of these data are their long follow-time from high-level cancer registries. Such long-term national data are available nowhere outside the Nordic countries. It should be pointed out that comparison of tumor stages in international studies is anyway problematic. For example, the TNM system used in many European cancer registries does not completely match the tumor grading “localized, regional, distant” used by SEER [53]. Similarly, the closely collaborating Nordic cancer registries cannot directly compare data on tumor characteristics (stage) [54].

In conclusion, the five female cancers showed different survival histories in the Nordic countries. Survival rates increased constantly for breast and ovarian cancers, they did also so, but more modestly, for endometrial and cervical cancers; for vulvar cancer, the trends were variable and country-specific. The overall survival development appeared to consist of small steps forward which could have been achieved by continuous improvements in treatment and early detection and overall organization of patient care, enabling sustained development without major break-throughs. However, as the present independent survival data extend only to year 2015 (see methods), it is too early to test the possible paradigm shift enabled by immunotherapy for endometrial and cervical cancers. The survival boost for ovarian cancer was probably achieved through the understanding of the importance of lymph nodal and peritoneal spread of tumor cells and delayed locoregional confinement of metastases. For cervical and vulvar cancers, the high early mortality requires attention and could be helped by increasing public awareness of early symptoms in these cancers and developing pathways for fast patient entrance to diagnostics and treatment.

## Data Availability

A publically available database was used.

## Abbreviations

CI: Confidence interval
DK: Denmark
FI: Finland
GAM: Gaussian generalized additive model
HPV: Human papilloma virus
IARC: International Agency for Research of Cancer
ICD: International Classification of Diseases
N: Number
NO: Norway
NOS: Not otherwise specified
PAF: Population attributable fractions
SE: Sweden
SEER: Surveillance, Epidemiology and End Results
TNM: Tumor-node-metastasis.

## Additional Points

*Key Message*. Survival in the Nordic countries has developed well for breast, endometrial and ovarian cancers while improvement for cervical and vulvar cancer has been modest. Country-specific differences suggest that a closer collaboration may help to improve the countries will lagging development.

## Supplementary Materials

Supplementary Materials Supplementary Table 1. 1-year (left part) and 5-year (right part) relative survival [95% confidence interval] in female-associated cancers from 1971 to 2020, across 4 Nordic countries. Cancer localizations include: (a) breast, (b) endometrium, (c) ovary, (d) *Cervix uteri* and (e) vulva. ^*∗*^Significant increase between the marked and the next period (95% confidence intervals do not overlap). Supplementary Table 2. 5/1-year (4-year conditional) survival in female-associated cancers from 1971 to 2020, across 4 Nordic countries. Cancer localizations include: (a) breast, (b) endometrium, (c) ovary, (d) *Cervix uteri* and (e) vulva.
