# Supplementary material for: Survival, Incidence, and Mortality Trends in Female Cancers in the Nordic Countries
Source: Obstet Gynecol Int. 2023 Jul 7;2023:6909414. doi: 10.1155/2023/6909414 (PMC10348860; doi:10.1155/2023/6909414)
Supplement: Supplementary Materials — Supplementary Table 1. 1-year (left part) and 5-year (right part) relative survival [95% confidence interval] in female-associated cancers from 1971 to 2020, across 4 Nordic countries. Cancer localizations include: (a) breast, (b) endometrium, (c) ovary, (d) Cervix uteri and (e) vulva. ∗Significant increase between the marked and the next period (95% confidence intervals do not overlap). Supplementary Table 2. 5/1-year (4-year conditional) survival in female-associated cancers from 1971 to 2020, across 4 Nordic countries. Cancer localizations include: (a) breast, (b) endometrium, (c) ovary, (d) Cervix uteri and (e) vulva. [file 6909414.f1.docx]

**Survival in female-associated cancers in the Nordic countries through a half century**

Filip Tichanek, Asta Försti, Otto Hemminki, Akseli Hemminki, Kari Hemminki

**Supplementary Table 1**. 1-year (left part) and 5-years (right part) relative survival [95% confidence interval] in female-associated cancers from 1971 to 2020, across 4 Nordic countries. Cancer localizations include: (a) breast, (b) endometrium, (c) ovary, (d) *Cervix uteri* and (e) vulva. *Significant increase between the marked and the next period (95% confidence intervals do not overlap).

| 1-year survival | | | | | 5-years survival |  |  |  |
| --- | --- | --- | --- | --- | --- | --- | --- | --- |
| (a) breast | Denmark | Finland | Norway | Sweden | Denmark | Finland | Norway | Sweden |
| 1971-1975 | 88.2 [87.3-89.0]* | 87.9 [86.7-89.2]* | 91.0 [90.1-92.0] | 90.8 [90.3-91.4]* | 61.2 [59.8-62.7]* | 59.3 [57.3-61.3]* | 65.0 [63.3-66.8] | 67.4 [66.4-68.4]* |
| 1976-1980 | 90.0 [89.3-90.7] | 90.6 [89.7-91.5] | 92.4 [91.6-93.2]* | 93.9 [93.5-94.4] | 66.1 [64.8-67.3] | 64.0 [62.5-65.5]* | 70.2 [68.6-71.7]* | 74.9 [74.0-75.8] |
| 1981-1985 | 90.1 [89.4-90.7]* | 92.1 [91.4-92.8]* | 94.0 [93.4-94.7] | 94.7 [94.3-95.1] | 66.6 [65.5-67.8]* | 71.4 [70.0-72.8]* | 73.2 [71.8-74.6] | 76.6 [75.7-77.5]* |
| 1986-1990 | 91.5 [90.9-92.1]* | 94.1 [93.5-94.7]* | 93.7 [93.0-94.3] | 95.2 [94.9-95.6] | 70.4 [69.3-71.5]* | 77.8 [76.5-79.1]* | 75.2 [73.9-76.5] | 80.0 [79.2-80.7]* |
| 1991-1995 | 92.9 [92.4-93.5] | 95.3 [94.8-95.9] | 94.2 [93.6-94.8]* | 95.9 [95.6-96.3]* | 74.5 [73.4-75.5]* | 80.5 [79.3-81.7]* | 76.8 [75.6-78.1]* | 82.8 [82.1-83.6]* |
| 1996-2000 | 93.7 [93.1-94.2]* | 95.6 [95.1-96.1] | 95.8 [95.3-96.3] | 96.7 [96.4-97.0] | 78.8 [77.8-79.8]* | 84.3 [83.3-85.3]* | 84.0 [82.9-85.1] | 85.5 [84.8-86.2] |
| 2001-2005 | 95.1 [94.6-95.5]* | 96.3 [95.8-96.7] | 96.4 [95.9-96.9] | 97.1 [96.8-97.4]* | 82.5 [81.6-83.4]* | 86.4 [85.5-87.4]* | 85.2 [84.2-86.3] | 86.7 [86.1-87.4]* |
| 2006-2010 | 96.1 [95.7-96.5]* | 96.6 [96.2-97.0]* | 96.6 [96.1-97.1] | 97.7 [97.5-98.0] | 86.2 [85.4-87.0]* | 88.4 [87.6-89.2]* | 86.5 [85.5-87.6]* | 89.6 [89.0-90.2]* |
| 2011-2015 | 97.3 [97.0-97.6] | 97.4 [97.1-97.8] | 97.5 [97.1-97.9] | 98.1 [97.8-98.3] | 89.6 [88.9-90.4] | 90.2 [89.5-90.9] | 89.9 [89.0-90.8] | 91.7 [91.1-92.2]* |
| 2016-2020 | 97.4 [97.1-97.7] | 97.4 [97.1-97.7] | 97.4 [97.0-97.8] | 98.3 [98.1-98.5] | 90.2 [89.5-90.9] | 90.8 [90.2-91.5] | 90.8 [90.0-91.7] | 92.3 [91.7-92.8] |
| (b) endometrium | | | | | | | | |
| 1971-1975 | 84.4 [82.4-86.4] | 83.6 [80.9-86.4] | 84.1 [81.3-87.1] | 85.3 [83.8-86.8]* | 66.3 [63.3-69.5] | 61.6 [57.8-65.7] | 70.5 [66.4-74.8] | 71.0 [68.7-73.3] |
| 1976-1980 | 85.0 [83.3-86.8] | 83.0 [80.8-85.2]* | 86.5 [84.4-88.7] | 88.1 [86.9-89.3] | 71.4 [68.9-74.1] | 65.9 [63.0-68.9]* | 73.0 [69.8-76.4] | 71.8 [69.7-73.8] |
| 1981-1985 | 86.2 [84.7-87.8] | 87.5 [85.8-89.3] | 87.7 [85.8-89.7] | 89.4 [88.3-90.6] | 72.9 [70.6-75.3] | 72.1 [69.4-74.9] | 70.9 [67.9-74.0] | 74.9 [72.9-76.8] |
| 1986-1990 | 88.3 [86.9-89.8] | 87.2 [85.7-88.9]* | 86.5 [84.7-88.3] | 91.1 [90.1-92.1] | 75.7 [73.4-78.0] | 73.5 [71.0-76.0]* | 72.0 [69.3-74.8] | 78.0 [76.3-79.8] |
| 1991-1995 | 89.9 [88.6-91.2] | 90.7 [89.4-92.1] | 88.6 [87.1-90.2] | 92.6 [91.7-93.4] | 77.9 [75.7-80.1] | 78.9 [76.6-81.2] | 76.5 [74.0-79.0] | 80.3 [78.8-81.8] |
| 1996-2000 | 91.3 [90.0-92.6] | 91.8 [90.7-93.0] | 91.4 [90.1-92.7] | 94.0 [93.2-94.7] | 81.2 [79.1-83.5] | 80.6 [78.6-82.7] | 79.5 [77.3-81.7] | 83.2 [81.8-84.6] |
| 2001-2005 | 91.2 [90.0-92.4] | 92.2 [91.2-93.2] | 92.4 [91.4-93.5] | 94.7 [94.1-95.4] | 79.8 [77.8-81.8] | 83.8 [82.1-85.6] | 82.0 [80.1-83.9] | 85.2 [83.9-86.4] |
| 2006-2010 | 92.1 [91.0-93.2] | 93.6 [92.7-94.5] | 93.2 [92.2-94.2] | 94.6 [94.0-95.2] | 83.6 [81.8-85.5] | 84.4 [82.8-86.0] | 83.8 [82.0-85.6] | 85.3 [84.1-86.5] |
| 2011-2015 | 94.0 [93.1-94.9] | 93.2 [92.3-94.1] | 94.0 [93.0-94.9] | 95.2 [94.6-95.8] | 84.3 [82.6-86.0] | 83.0 [81.5-84.5] | 84.8 [83.2-86.5] | 85.6 [84.5-86.8] |
| 2016-2020 | 93.5 [92.6-94.4] | 92.8 [91.9-93.6] | 94.8 [94.0-95.6] | 95.0 [94.4-95.6] | 83.4 [81.8-85.0] | 82.6 [81.2-84.1] | 85.9 [84.4-87.4] | 86.1 [85.0-87.3] |
| (c) ovary | | | | | | | | |
| 1971-1975 | 45.6 [43.3-48.0] | 54.0 [50.5-57.7] | 56.9 [53.9-59.9] | 55.7 [54.1-57.4]* | 20.8 [18.9-22.9] | 26.4 [23.1-30.1] | 29.7 [26.7-33.0] | 33.4 [31.7-35.1] |
| 1976-1980 | 48.2 [46.1-50.3]* | 54.1 [51.4-57.0]* | 60.8 [58.1-63.7] | 59.5 [57.8-61.1]* | 22.1 [20.4-24.0] | 25.8 [23.4-28.5] | 32.7 [30.0-35.8] | 34.8 [33.1-36.5] |
| 1981-1985 | 53.8 [51.8-55.8] | 59.8 [57.4-62.4] | 62.9 [60.6-65.3] | 65.7 [64.2-67.3]* | 24.2 [22.5-26.1] | 30.2 [27.9-32.6] | 33.3 [31.0-35.9] | 37.9 [36.2-39.6] |
| 1986-1990 | 58.7 [56.7-60.7]* | 61.8 [59.5-64.2]* | 61.8 [59.6-64.0]* | 68.9 [67.4-70.5]* | 27.9 [26.1-29.9] | 31.8 [29.5-34.2] | 32.2 [30.1-34.5] | 39.3 [37.6-41.0] |
| 1991-1995 | 62.9 [60.9-64.9] | 66.8 [64.6-69.0] | 67.4 [65.3-69.6]* | 72.2 [70.7-73.7]* | 27.5 [25.7-29.5]* | 33.2 [31.0-35.5]* | 35.1 [32.9-37.4]* | 38.8 [37.1-40.5]* |
| 1996-2000 | 64.4 [62.5-66.4]* | 70.7 [68.6-72.8]* | 72.5 [70.5-74.5]* | 78.5 [77.0-79.9] | 32.8 [31.0-34.8] | 40.7 [38.4-43.1] | 39.7 [37.5-42.0] | 42.7 [41.0-44.6] |
| 2001-2005 | 70.8 [68.9-72.7] | 76.0 [74.2-77.9] | 77.0 [75.1-78.9] | 81.1 [79.7-82.5] | 34.6 [32.6-36.6]* | 43.4 [41.2-45.7] | 41.6 [39.4-44.0] | 44.3 [42.5-46.1]* |
| 2006-2010 | 74.1 [72.3-75.9]* | 78.3 [76.5-80.0] | 77.0 [75.2-78.9] | 83.3 [82.0-84.7]* | 40.0 [38.0-42.1] | 44.4 [42.3-46.7] | 44.7 [42.5-47.1] | 46.2 [44.4-48.1]* |
| 2011-2015 | 78.5 [76.8-80.1] | 78.8 [77.2-80.5] | 80.1 [78.3-82.0]* | 86.6 [85.3-88.0] | 42.6 [40.6-44.7] | 44.0 [42.0-46.1] | 48.6 [46.3-51.0]* | 51.6 [49.6-53.6] |
| 2016-2020 | 81.6 [80.0-83.2] | 81.2 [79.6-82.8] | 85.3 [83.6-87.0] | 88.4 [87.1-89.7] | 43.9 [41.9-46.0] | 46.6 [44.5-48.8] | 52.4 [50.1-54.9] | 52.9 [51.0-54.9] |
| (d) *Cervix uteri* | | | | | | | | |
| 1971-1975 | 83.1 [81.6-84.6] | 85.6 [83.4-87.8] | 85.1 [83.1-87.2] | 83.2 [81.8-84.7] | 59.0 [57.0-61.1] | 61.7 [58.7-64.9] | 65.9 [63.1-68.8] | 61.4 [59.4-63.4] |
| 1976-1980 | 82.6 [81.1-84.1] | 84.4 [82.0-86.8] | 84.6 [82.7-86.5] | 84.3 [82.9-85.8] | 60.1 [58.1-62.2] | 59.9 [56.7-63.3] | 64.8 [62.3-67.5] | 61.1 [59.1-63.3] |
| 1981-1985 | 81.1 [79.6-82.7] | 87.5 [85.3-89.7] | 85.3 [83.3-87.3] | 85.6 [84.2-87.0] | 59.4 [57.4-61.6] | 61.4 [58.0-65.0] | 65.4 [62.8-68.2] | 63.9 [61.8-66.0] |
| 1986-1990 | 83.8 [82.2-85.3] | 82.0 [79.2-84.9] | 85.1 [83.2-87.1] | 85.2 [83.7-86.8] | 62.3 [60.2-64.5] | 56.5 [52.7-60.6] | 62.3 [59.6-65.1] | 64.8 [62.6-67.0] |
| 1991-1995 | 85.2 [83.6-86.8] | 84.7 [82.1-87.4] | 88.2 [86.5-90.1] | 86.3 [84.8-87.9] | 64.7 [62.5-67.1] | 64.3 [60.6-68.3] | 67.1 [64.4-69.9] | 65.4 [63.3-67.7] |
| 1996-2000 | 83.8 [82.0-85.7] | 88.1 [85.6-90.6] | 85.9 [83.8-88.0] | 85.8 [84.2-87.5] | 64.1 [61.6-66.7] | 67.8 [64.1-71.7] | 67.1 [64.2-70.1] | 64.8 [62.5-67.3] |
| 2001-2005 | 82.7 [80.7-84.7] | 86.0 [83.2-88.9] | 87.8 [85.7-89.9] | 86.4 [84.8-88.0] | 64.3 [61.7-67.1] | 65.8 [61.9-70.1] | 71.4 [68.5-74.4] | 67.2 [64.9-69.6] |
| 2006-2010 | 85.1 [83.1-87.1] | 86.6 [83.7-89.6] | 88.1 [86.1-90.2] | 87.7 [86.1-89.2] | 67.9 [65.2-70.7] | 65.3 [61.1-69.7] | 71.4 [68.4-74.5] | 69.0 [66.7-71.3] |
| 2011-2015 | 88.1 [86.3-90.0] | 86.2 [83.5-89.0] | 89.2 [87.1-91.4] | 88.0 [86.5-89.6] | 71.6 [69.0-74.3] | 67.1 [63.3-71.1] | 73.0 [70.0-76.2] | 69.5 [67.2-71.8]* |
| 2016-2020 | 90.1 [88.4-91.8] | 86.4 [83.7-89.2] | 91.0 [89.3-92.8] | 90.3 [89.0-91.7] | 75.4 [72.9-78.0] | 69.7 [66.1-73.6] | 75.6 [72.8-78.6] | 74.5 [72.3-76.6] |
| (e) vulva | | | | | | | | |
| 1971-1975 | 84.2 [80.1-88.6] | 69.9 [63.9-76.6] | 82.1 [76.9-87.7] | 80.0 [76.5-83.7] | 71.0 [64.9-77.7] | 45.4 [38.5-53.5] | 64.4 [56.7-73.2] | 59.6 [54.5-65.1] |
| 1976-1980 | 83.7 [79.9-87.7] | 75.9 [70.3-81.9] | 82.3 [77.3-87.6] | 81.6 [78.3-85.2]* | 60.6 [54.9-66.8]* | 52.0 [45.3-59.7] | 64.2 [57.4-71.9] | 62.4 [57.7-67.6] |
| 1981-1985 | 84.8 [80.7-89.1] | 80.4 [75.7-85.3] | 80.4 [75.4-85.8] | 86.2 [83.4-89.0] | 71.3 [65.3-77.9] | 56.4 [50.0-63.6] | 63.3 [56.5-71.0] | 59.6 [55.1-64.4] |
| 1986-1990 | 83.4 [79.0-87.9] | 81.5 [76.7-86.7] | 82.2 [77.7-87.0] | 83.2 [80.2-86.3] | 67.1 [60.8-74.0] | 59.2 [52.0-67.4] | 61.8 [54.9-69.5] | 62.2 [57.5-67.2] |
| 1991-1995 | 86.5 [82.4-90.8] | 84.9 [80.4-89.5] | 84.9 [80.5-89.6] | 85.6 [82.6-88.8] | 68.1 [61.7-75.1] | 64.5 [57.9-71.8] | 66.2 [59.7-73.3] | 64.6 [60.2-69.3] |
| 1996-2000 | 87.3 [83.5-91.4] | 78.9 [74.3-83.9]* | 86.7 [81.8-91.9] | 83.3 [80.3-86.3] | 69.8 [63.8-76.4] | 65.0 [59.0-71.7] | 61.1 [53.5-69.9]* | 62.2 [58.0-66.7]* |
| 2001-2005 | 85.4 [81.4-89.6] | 84.1 [80.2-88.1] | 82.9 [78.6-87.5]* | 86.3 [83.5-89.2] | 67.1 [61.2-73.7] | 68.5 [62.9-74.6] | 70.3 [64.4-76.7] | 68.2 [64.1-72.5] |
| 2006-2010 | 86.2 [82.8-89.7] | 83.6 [79.7-87.8] | 88.9 [85.4-92.6] | 86.5 [83.9-89.2]* | 70.2 [65.0-75.9] | 67.9 [62.6-73.7] | 67.8 [62.1-74.0] | 67.1 [63.2-71.2]* |
| 2011-2015 | 86.3 [83.1-89.7] | 85.5 [82.2-89.0] | 87.2 [83.6-91.1] | 90.7 [88.6-92.7] | 69.3 [64.5-74.5] | 71.3 [66.6-76.4] | 67.9 [62.2-74.2] | 74.9 [71.4-78.5] |
| 2016-2020 | 88.5 [85.7-91.4] | 87.3 [84.3-90.5] | 87.3 [84.0-90.7] | 87.6 [85.4-89.9] | 72.0 [67.5-76.7] | 70.6 [65.8-75.7] | 71.5 [66.5-77.0] | 72.9 [69.6-76.3] |

**Supplementary Table 2**. 5/1-year (4-years conditional) survival in female-associated cancers from 1971 to 2020, across 4 Nordic countries. Cancer localizations include: (a) breast, (b) endometrium, (c) ovary, (d) *Cervix uteri* and (e) vulva.

| 5-years/1-year (4-years conditional) survival | | | | |
| --- | --- | --- | --- | --- |
| (a) breast | Denmark | Finland | Norway | Sweden |
| 1971-1975 | 69.4 | 67.5 | 71.4 | 74.2 |
| 1976-1980 | 73.4 | 70.6 | 76.0 | 79.8 |
| 1981-1985 | 73.9 | 77.5 | 77.9 | 80.9 |
| 1986-1990 | 76.9 | 82.7 | 80.3 | 84.0 |
| 1991-1995 | 80.2 | 84.5 | 81.5 | 86.3 |
| 1996-2000 | 84.1 | 88.2 | 87.7 | 88.4 |
| 2001-2005 | 86.8 | 89.7 | 88.4 | 89.3 |
| 2006-2010 | 89.7 | 91.5 | 89.5 | 91.7 |
| 2011-2015 | 92.1 | 92.6 | 92.2 | 93.5 |
| 2016-2020 | 92.6 | 93.2 | 93.2 | 93.9 |
| (b) endometrium | | | | |
| 1971-1975 | 78.6 | 73.7 | 83.8 | 83.2 |
| 1976-1980 | 84.0 | 79.4 | 84.4 | 81.5 |
| 1981-1985 | 84.6 | 82.4 | 80.8 | 83.8 |
| 1986-1990 | 85.7 | 84.3 | 83.2 | 85.6 |
| 1991-1995 | 86.7 | 87.0 | 86.3 | 86.7 |
| 1996-2000 | 88.9 | 87.8 | 87.0 | 88.5 |
| 2001-2005 | 87.5 | 90.9 | 88.7 | 90.0 |
| 2006-2010 | 90.8 | 90.2 | 89.9 | 90.2 |
| 2011-2015 | 89.7 | 89.1 | 90.2 | 89.9 |
| 2016-2020 | 89.2 | 89.0 | 90.6 | 90.6 |
| (c) ovary | | | | |
| 1971-1975 | 45.6 | 48.9 | 52.2 | 60.0 |
| 1976-1980 | 45.9 | 47.7 | 53.8 | 58.5 |
| 1981-1985 | 45.0 | 50.5 | 52.9 | 57.7 |
| 1986-1990 | 47.5 | 51.5 | 52.1 | 57.0 |
| 1991-1995 | 43.7 | 49.7 | 52.1 | 53.7 |
| 1996-2000 | 50.9 | 57.6 | 54.8 | 54.4 |
| 2001-2005 | 48.9 | 57.1 | 54.0 | 54.6 |
| 2006-2010 | 54.0 | 56.7 | 58.1 | 55.5 |
| 2011-2015 | 54.3 | 55.8 | 60.7 | 59.6 |
| 2016-2020 | 53.8 | 57.4 | 61.4 | 59.8 |
| (d) *Cervix uteri* | | | | |
| 1971-1975 | 71.0 | 72.1 | 77.4 | 73.8 |
| 1976-1980 | 72.8 | 71.0 | 76.6 | 72.5 |
| 1981-1985 | 73.2 | 70.2 | 76.7 | 74.6 |
| 1986-1990 | 74.3 | 68.9 | 73.2 | 76.1 |
| 1991-1995 | 75.9 | 75.9 | 76.1 | 75.8 |
| 1996-2000 | 76.5 | 77.0 | 78.1 | 75.5 |
| 2001-2005 | 77.8 | 76.5 | 81.3 | 77.8 |
| 2006-2010 | 79.8 | 75.4 | 81.0 | 78.7 |
| 2011-2015 | 81.3 | 77.8 | 81.8 | 79.0 |
| 2016-2020 | 83.7 | 80.7 | 83.1 | 82.5 |
| (e) vulva | | | | |
| 1971-1975 | 0.84 | 0.65 | 0.78 | 0.75 |
| 1976-1980 | 0.72 | 0.69 | 0.78 | 0.76 |
| 1981-1985 | 0.84 | 0.70 | 0.79 | 0.69 |
| 1986-1990 | 0.80 | 0.73 | 0.75 | 0.75 |
| 1991-1995 | 0.79 | 0.76 | 0.78 | 0.75 |
| 1996-2000 | 0.80 | 0.82 | 0.70 | 0.75 |
| 2001-2005 | 0.79 | 0.81 | 0.85 | 0.79 |
| 2006-2010 | 0.81 | 0.81 | 0.76 | 0.78 |
| 2011-2015 | 0.80 | 0.83 | 0.78 | 0.83 |
| 2016-2020 | 0.81 | 0.81 | 0.82 | 0.83 |
